# Trends in research related to menopausal hormone therapy from 2000 to 2021: A bibliometric analysis

**DOI:** 10.3389/fmed.2022.952487

**Published:** 2022-10-28

**Authors:** Jing Li, Zhipeng Wei, Jingxi Wu, Kaili Min, Xiao Li, Yuan Yao, Yao Li, Ningning Zhang, Anya Shi, Jiani Han, Chengdong Qiao, Kehu Yang

**Affiliations:** ^1^Evidence-Based Social Sciences Research Center/Health Technology Assessment Center, School of Public Health, Lanzhou University, Lanzhou, China; ^2^Evidence-Based Medicine Center, School of Basic Medical Sciences, Lanzhou, China; ^3^Key Laboratory of Evidence Based Medicine and Knowledge Translation of Gansu Province, Lanzhou, China; ^4^First Clinical Medical College of Lanzhou University, Lanzhou, China; ^5^Second Clinical Medical College of Lanzhou University, Lanzhou, China; ^6^The First Hospital of Lanzhou University, Lanzhou, China

**Keywords:** menopausal hormone therapy, menopausal women, treatment, adverse events, bibliometric analysis

## Abstract

We conducted the present bibliometric analysis to explore menopausal hormone therapy (MHT)-related research trends between 2000 and 2021. The Web of Science database was systematically searched from 2000 to 2021 to retrieve MHT-related publications. Visualization mapping and keyword cluster graphs were utilized to illustrate the research topics and hotpots. We included 11,616 MHT-related publications for this bibliometric analysis. The results showed that (1) MHT-related research had a very slow increase in the past 22 years, and the trend fluctuated. Sum of times cited and average citations per item had the same trend: a sharp decline from 2002 to 2003, and a rapid increase from 2003 to 2006, reaching the peak in 2006, then following a downward trend. The average H-index was 57, peaking in 2001; (2) the USA, the League of European Research Universities, and Dr. JoAann Manson from Harvard University contributed the most; (3) Menopause: The Journal of The North American Menopause Society had the most significant number of MHT-related publications; (4) the research hotpots primarily focused on MHT for treating menopausal symptoms and the impact of MHT on women’s health. According to previous studies, MHT was the most effective treatment for managing vasomotor symptoms of menopause, but results from the clinical trials and observational studies regarding MHT adverse events remain inconsistent. Mechanisms are fundamental when clinical studies give conflicting results. Therefore, future studies should focus on adverse events and their mechanisms.

## Introduction

Menopause is often associated with various unpleasant physical and mental symptoms, such as hot flashes and night sweats, insomnia, anxiety, depression, decreased libido, vaginal dryness, or difficulty concentrating. About 80% of menopausal women are affected by such symptoms; hot flashes and night sweats account for approximately 70% of midlife women (1). These symptoms have a significant adverse effect on daily functioning and the quality of life (2, 3). Clinical guidelines indicate that vasomotor symptoms of menopause (VMS) typically last from 6 months to 2 years. However, new researches suggest that for many women, the duration of symptoms could persist for a decade or even longer (1, 4).

Menopausal hormone therapy (MHT) has been utilized for over 50 years to treat menopausal women with hot flashes and other menopausal symptoms. MHT is the most effective treatment for managing VMS and improves the quality of life (5, 6). The two main hormones used in MHT are estrogen and progestogen, of which the latter was only used for women having a uterus. The two hormones usually come in several forms, like tablets, skin patches, estrogen gel, implants, and other types (7). With the use of MHT, thousands of published manuscripts have reported mechanistic (8, 9), therapeutic (10, 11), and preventive studies (9, 12). As for adverse events of MHT for menopausal women, the results from the clinical trials and observational studies regarding MHT adverse events remain inconsistent: the largest randomized controlled trial of MHT, Women’s Health Initiative (WHI), showed that compared with placebo, conjugated equine estrogen elevated the risk of dementia (13), breast cancer (14), and stroke (15). However, some observational studies reported a reduced risk of Alzheimer’s disease and all-cause dementia in users of MHT (11, 16–18), and utilizing exogenous estrogens was cardio-protective for post-menopausal women. Based on the conflicts, mechanisms are essential to understand complex systems (e.g., physiological or social systems) and can help us explain, predict, and intervene (19). Researchers may conduct horizontal and longitudinal studies to explore the adverse events and MHT-related mechanisms. It is ideal for clinicians to treat menopausal women after fully understanding MHT-related studies.

Bibliometric analysis is a statistical method used to analyze and visualize critical characteristics from published articles and identify research trends in a specific field through online literature databases (20–24). Our objective is to gain an enhanced understanding of existing research hotspots and potential trends and to provide appropriate reference guidelines for future research.

## Methods and analysis

### Data source and search strategy

Using the keywords that related to MHT: “Hormone Replacement Therapy,” “Estrogen Replacement Therapy,” “estrogen*,” “progestin*,” “medroxyprogesterone,” “hormone therapy,” “menopausal hormone therapy,” “HRT,” “ERT,” “progestins,” “medroxyprogesterone acetate,” “dydrogesterone,” “norethisterone,” “norethindrone,” “estrogen,” “estrogen,” “conjugated equine estrogen,” “CEE,” “premarin,” “estriol,” “estradiol,” “estradiol*”; and menopause-related keywords including “menopause,” “climacteric,” “postmenopause,” “post-menopause*,” “post menopause*,” “perimenopausal,” “premenopause,” and “perimenopause.” We screened the Web of Science Core Collection (WOSCC) (including the Science Citation Index Expanded, Social Sciences Citation Index, and Arts & Humanities Citation Index) from January 2000 to December 2021 to identify publications associated with MHT. The details of search terms are shown in Supplementary Table 1. As the metrics keep changing over time, all the searches and data exports were completed on the same day to avoid the possible bias due to frequent updates of the database.

### Inclusion and exclusion criteria

We included or excluded studies based on the classification of WOSCC, and the filters of WOSCC screened the articles.

We included articles that met the following inclusion criteria: (1) articles published between 2010 and 2021, (2) articles regarding MHT-related research, and (3) articles with basic information.

**FIGURE 1:**
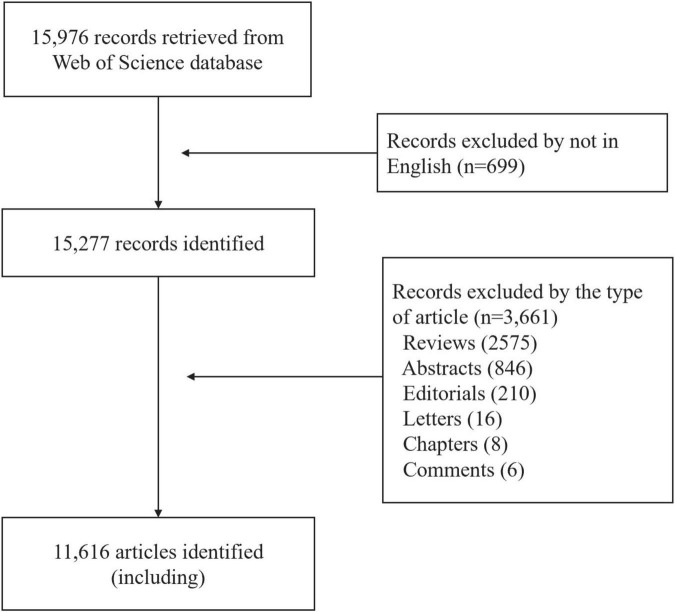
Flow diagram of study selection of menopausal hormone therapy (MHT)-related research.

The following documents were excluded: (1) non-English publications; and (2) reviews, abstracts, letters, editorials, and books. The screening process is illustrated in Figure 1.

### Data collection and cleaning

We retrieved (1) the characteristics of all the retrieved publications; (2) the 2020 journal impact factor (IF) (25), 5-year IF (25), and publication counts of the journals; (3) the number of publications each year, Hirsch index (h-index), various citation values [average citations per item (ACPI), sum of times cited (STC) and the number of citations of most-cited item (NCMCI)]; (4) the top-5 most-publications research areas (top-5 research areas) of the institutions having more than 200 MHT-related publications; and (5) h-index, various citation values, and the top-5 research areas of the top-10 most-publications authors (top-10 authors). All the documents were downloaded in a tab separator format.

We standardized the keywords with the same meaning but in different styles: “climacteric” was replaced by “menopause,” “breast carcinoma” by “breast cancer,” “heart disease” by “cardiovascular disease,” “BMD” by “bone mineral density,” “BMI” by “body mass index,” “hot flush,” and “hot flash” by “hot flashes.”

### Statistical analysis

We used Microsoft Excel 2019 (Washington, DC, USA) and VOSviewer (Version 1.6.18, Leiden University, The Netherlands) for data processing and visualizing the publication characteristics (26). VOS viewer (1.6.18) includes three types of maps—network, coverage, and density visualization. It can help visualize the co-author analysis of countries/institutions/authors, co-citation analysis of journals/references, literature citation analysis, and keywords co-occurrence analysis (20). Keywords co-occurrence can effectively reflect the research hotspots in the discipline fields, with auxiliary support for scientific research (27, 28).

## Results

### Publication characteristics

**FIGURE 2:**
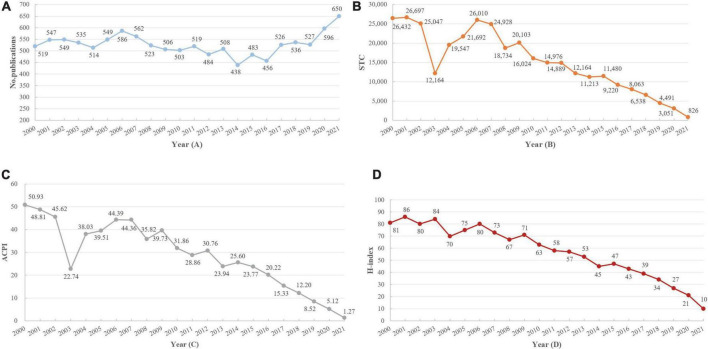
The annual number of menopausal hormone therapy (MHT)-related publications **(A)**, STC **(B)**, ACPI **(C)**, and H-index **(D)**.

We identified 15,976 records from 2000 to 2021. Based on the inclusion and exclusion criteria, our study included 11,616 articles. Figure 2A plots the annual number of MHT-related publications from 2000 to 2021, MHT-related research showed a very slow increase in the past 22 years, and the trend fluctuated. The publications ranged from 438 to 650. A slow downward trend was observed between 2006 and 2014; the lowest was in 2014 (438), then gradually rose from 2015 to 2021, peaking at 650 in 2021. These indicate that the research heat of MHT fluctuated and progressively increased in recent years. Additionally, we found that the STC (Figure 2B), ACPI (Figure 2C), and H-index (Figure 2D) for MHT generally declined. As for STC and ACPI, there was a sharp decline from 2002 to 2003 and a rapid rise from 2003 to 2006, reaching the peak in 2006 (STC, 26,010; ACPI, 44.39) and then kept a downward trend, achieving the lowest point in 2021 (STC, 826; ACPI 1.27). The average H-index was 57, peaking in 2001 (86) (Figure 2D). These data showed significant progress in MHT field over the last two decades, especially between 2000 and 2003.

### The distribution of publications by journal

A total of 2,044 journals were included in MHT-related research during the study period. Table 1 shows the journals with more than 100 MHT-related publications. Over the past 22 years, Menopause-The Journal of The North American Menopause Society (1,070) showed the highest number of publications, followed by Maturitas (758) and Climacteric (499). In addition, 2020 IF ranged from 2.26 (Gynecological Endocrinology) to 7.329 (Fertility and Sterility). The 5-year IF ranged from 2.096 (Gynecological Endocrinology) to 7.366 (Human Reproduction). Moreover, the Journal of Clinical Endocrinology and Metabolism depicted the highest ACPI and H-index, indicating the higher quality of manuscripts published in this journal.

**TABLE 1:** Journals with more than 100 menopausal hormone therapy (MHT)-related publications (*N* = 11,616).

Journal	No. publications	IF (2020)	5-year IF	STC	ACPI	H-index
Menopause: The Journal of The North American Menopause Society	1,070	2.953	3.206	32,615	30.48	83
Maturitas	758	4.342	4.877	18,397	24.27	61
Climacteric	499	3.005	3.207	10,713	21.47	48
Gynecological Endocrinology	265	2.260	2.096	3,423	12.92	27
Journal of Clinical Endocrinology and Metabolism	198	5.958	6.793	13,320	67.27	61
Fertility and Sterility	176	7.329	7.253	7,284	41.39	47
Breast Cancer Research and Treatment	134	4.872	4.698	5,331	39.78	37
Human Reproduction	120	6.918	7.366	4,758	39.65	41
Osteoporosis International	119	4.507	4.802	4,046	34.00	37
PLOS ONE	107	3.240	3.788	2,052	19.18	26

IF, impact factor; ACPI, average citations per item; STC, sum of times cited.

### Distribution of publications by country

**FIGURE 3:**
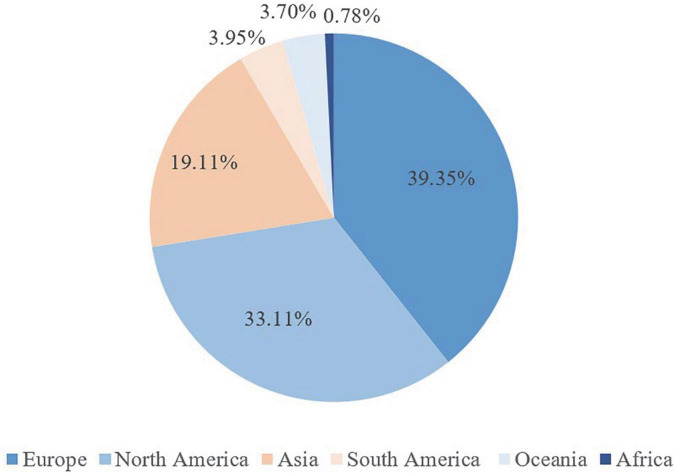
Proportion of menopausal hormone therapy (MHT)-related publications in terms of regions.

**FIGURE 4:**
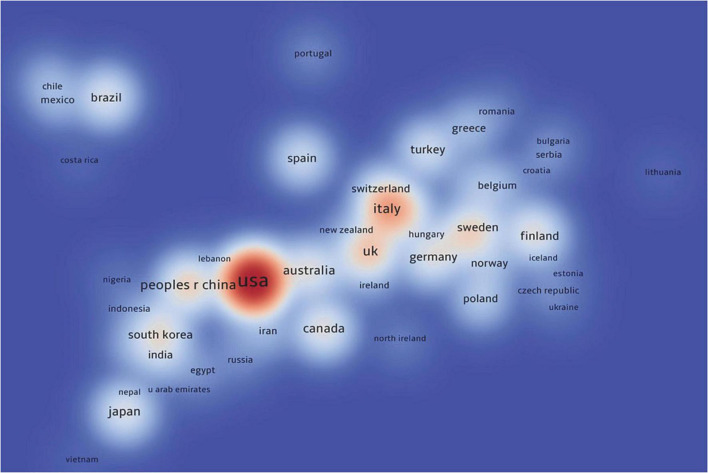
Map of Countries that published menopausal hormone therapy (MHT)-related publications.

A total of 117 countries published MHT-related studies, of which 39.35% were from Europe, 33.11% from North America, and 19.11% from Asia (Figure 3). The density map included 76 countries, with a frequency ≥ 5 times (Figure 4). The figure showed that the top 10 countries were the United States of America (USA) with 4,373, followed by Italy (944), the United Kingdom (UK) (809), China (699), Canada (547), Australia (505), Japan (479), Germany (466), France (451, 4%), and Sweden (425) (Figure 4 and Table 2). Among the top-10 countries are 1/2 from Europe, 1/5 from North America, 1/5 from Asia, and 1/10 from Oceania (Table 2).

**TABLE 2:** Characteristics of the top-10 countries (*N* = 11,616).

ID	Country	No. publications (%)	Region	H-index	ACPI	STC	NCMCI	Top-5 research areas
1	USA	4,374 (37.66)	North America	174	42.11	184,225	1,222	
								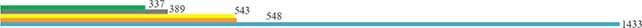
2	Italy	944 (8.13)	Europe	86	33.34	31,477	497	
								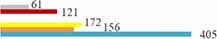
3	UK	809 (6.97)	Europe	92	42.82	34,644	914	
								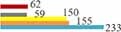
4	China	699 (6.02)	Asia	49	16.62	11,619	478	
								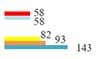
5	Canada	547 (4.71)	North America	75	39.25	21,468	873	
								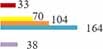
6	Australia	505 (4.35)	Oceania	71	44.8	22,622	873	
								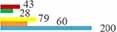
7	Japan	479 (4.12)	Asia	57	28.28	13,547	873	
								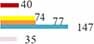
8	Germany	466 (4.01)	Europe	67	36.38	16,951	873	
								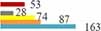
9	France	451 (3.88)	Europe	73	41.64	18,779	873	
								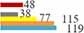
10	Sweden	425 (3.66)	Europe	62	37.01	15,729	497	
								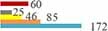

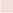
 Cardiovascular system cardiology; 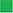 Medicine general Internal; 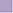 Neurosciences neurology; 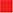 Public Environmental occupational health; 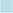 Research experimental medicine; 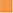 Pharmacology pharmacy; 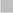 Oncology; 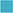 Reproductive biology; 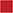 Obstetrics gynaecology; 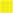 Geriatrics Gerontology; 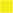 Endocrinology Metabolism.

ACPI, average citations per item; STC, sum of times cited; NCMCI, number of citations of most-cited item.

In terms of STC, h-index, and NCMCI among the top-10 countries, the USA contributed the most with the highest h-index (174), STC (184,255), and NCMCI (1,222), followed by the UK in h-index (92), STC (34,644), and NCMCI (914) (Table 2). As for ACPI, Australia showed the highest ACPI (44.8), followed by the UK (42.82), the USA (42.11), and France (41.64) (Table 2).

Among 11,616 MHT-related publications, 98 research areas were included. While addressing the top-5 research areas among top-10 countries, obstetrics-gynecology, endocrinology metabolism, and oncology areas were studied by all 10 countries. The USA published 1,433 MHT-related studies in the obstetrics-gynecology research area, then followed by Italy (405), the UK (233), Australia (200), and Sweden (172). Regarding endocrinology metabolism and oncology research areas, the USA had the largest publications, 543 and 548. Eight countries studied the geriatrics gerontology area except for USA and China. Among these eight countries, Italy had the most significant number of publications (121), then followed by the UK (62) and Sweden (60). The public environmental occupational health area was studied by the USA (389), UK (59), Germany (28), France (38), and Sweden (25) (Table 2).

### Distribution of publications by institution

Table 3 shows the 13 institutions with more than 200 MHT-related publications. Among them, 9/13 were from the USA. League of European Research Universities (LERU) was the top-1 most publications (767), followed by the University of California System (616), Harvard University (561), Pennsylvania Commonwealth System of Higher Education (PCSHE) (343), Brigham and Women’s Hospital (303), and National Institutes of Health (NIH) (301). As for STC, the USA was at the top-1 with 36,565 citations, followed by Harvard University (35,553) and the University of California System (34,022). We observed that three institutions with an H-index higher than 90 were the University of California System (97), Harvard University (94), and LERU (93).

**TABLE 3:** Institutions having more than 200 menopausal hormone therapy (MHT)-related publications (*N* = 11,616).

Institution	No. publications, *n* (%)	Country	H-index	ACPI	STC	Top-5 research areas
League of European Research Universities	767 (6.60%)	Multi-country*	93	47.67	36,565	1. Obstetrics Gynecology 2.Oncology 3. Endocrinology Metabolism 4. Geriatrics Gerontology 5. Public Environmental Occupational Health
University of California System	616 (5.30%)	USA	97	55.23	34,022	1. Obstetrics Gynecology 2. Oncology 3. Endocrinology Metabolism 4. Public Environmental Occupational Health 5. General Internal Medicine
Harvard University	561 (4.83%)	USA	94	63.37	35,553	1. Obstetrics Gynecology 2. Oncology 3. Endocrinology Metabolism 4. Public Environmental 5. Occupational Health 6. General Internal Medicine
Pennsylvania Commonwealth System of Higher Education	343 (2.95%)	USA	77	64.83	22,238	1. Obstetrics Gynecology 2. Endocrinology Metabolism 3. Public Environmental Occupational Health 4. Oncology 5. General Internal Medicine
Brigham and Women’s Hospital	303 (2.61%)	USA	71	64.50	19,542	1. Obstetrics Gynecology 2. Oncology 3. Public Environmental Occupational Health 4. Neurosciences Neurology 5. Endocrinology Metabolism
National Institutes of Health	301 (2.59%)	USA	68	63.19	19,019	1. Oncology 2. Obstetrics Gynecology 3. Public Environmental Occupational Health 4. Endocrinology Metabolism 5. Neurosciences Neurology
University of Pittsburgh	291 (2.51%)	USA	72	63.75	18,551	1. Obstetrics Gynecology 2. Endocrinology Metabolism 3. Public Environmental Occupational Health 4. Oncology 5. Cardiovascular System Cardiology
University of Southern California	213 (1.83%)	USA	50	46.87	9,983	1. Obstetrics Gynecology 2. Oncology 3. Public Environmental Occupational Health 4. Neurosciences Neurology 5. Endocrinology Metabolism
Inserm	212 (1.83%)	France	52	40.72	8,633	1. Oncology 2. Obstetrics Gynecology 3. Endocrinology Metabolism 4. Public Environmental Occupational Health 5. Neurosciences Neurology
University of California Los Angeles	212 (1.83%)	USA	62	60.46	12,818	1. Obstetrics Gynecology 2. Oncology 3. Endocrinology Metabolism 4. Public Environmental Occupational Health 5. General Internal Medicine
Udice French Research Universities	211 (1.82%)	France	52	40.71	8,590	1. Oncology 2. Obstetrics Gynecology 3. Endocrinology Metabolism Public Environmental 4. Occupational Health 5. Reproductive Biology
University of London	204 (1.76%)	UK	44	41.87	8,542	1. Oncology 2. Obstetrics Gynecology 3. Endocrinology Metabolism Public Environmental 4. Occupational Health 5. General Internal Medicine
University of Washington	203 (1.75%)	USA	51	47.95	9,733	1. Obstetrics Gynecology 2. Oncology 3. General Internal Medicine 4. Public Environmental Occupational Health 5. Endocrinology Metabolism

*Represent the twelve countries: UK, France, Germany, Italy, Sweden, Spain, Denmark, Finland, Belgium, Netherlands, Ireland, and Switzerland.

**FIGURE 5:**
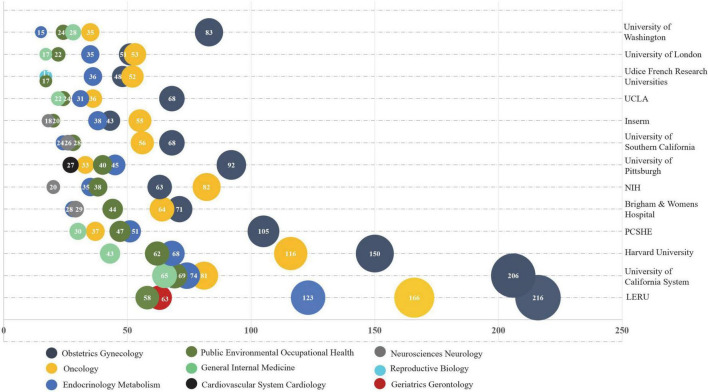
No. publications of top-5 research areas for the institutions with more than 200 menopausal hormone therapy (MHT)-related publications.

We analyzed the top five research areas among the 13 institutions with more than 200 MHT-related publications. These 13 institutions mainly focused on obstetrics, gynecology, oncology, endocrinology metabolism, public environmental occupational health, and general internal medicine (Figure 5).

### The distribution of publications by author

**FIGURE 6:**
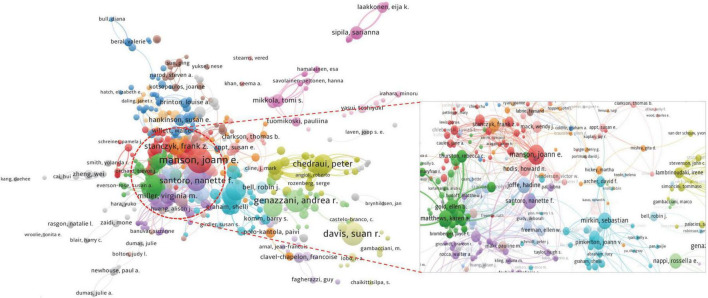
Co-authorship network of menopausal hormone therapy (MHT)-related publications.

A total of 47,559 authors were included in MHT-related publications, 611 authors with a frequency ≥ 5 times were incorporated in the collaboration network analysis, and 31 clusters were created. Dr. JoAann Manson (USA), Dr. Susan Davis (Australia), Dr. Andrea Genazzani (Italy), and Dr. Nanette Santoro (USA) had the highest number of publications. They were distributed in different clusters, showing close cooperative relationships with other authors (Figure 6).

**FIGURE 7:**
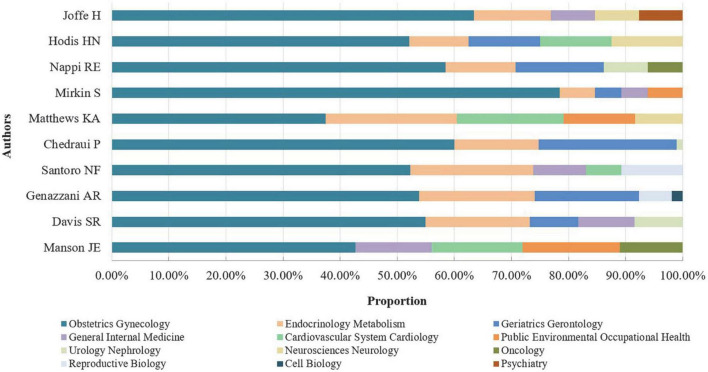
Proportion of top-5 research areas for the top-10 authors.

The top-10 authors with the most MHT-related publications mainly originated from the USA (3/5) and Italy (1/5) and were focused on Harvard University (1/5) and the University of Pisa (1/5) (Table 4). Most of the top-10 authors primarily focused on obstetrics gynecology, endocrinology metabolism, geriatrics gerontology, and seven research areas (Figure 7 and Table 4). The most-contributed research area of the top 10 authors was obstetrics gynecology (Figure 7).

**TABLE 4:** Characteristics of the top-10 authors (*N* = 11,616).

ID	Author	Country	No. publications	H-index	ACPI	STC	NCMCI	Institution	Department	Top-5 research areas
1	Dr. JoAann Manson	USA	90	41	90.77	8,169	1,175	Harvard University	Brigham and Women’s Hosp., Sch. Med., Div. Prevent. Med., Boston	Obstetrics Gynecology; Endocrinology Metabolism; Geriatrics Gerontology; Reproductive Biology; Cell Biology
2	Dr. Susan Davis	Australia	66	29	63.48	4,190	621	Monash University	Mayne Hlth. Dorevitch Pathol., Fairfield	Obstetrics Gynecology; Endocrinology Metabolism; General Internal Medicine; Geriatrics Gerontology; Urology Nephrology
3	Dr. Andrea Genazzani	Italy	62	28	30.61	1,898	165	University of Pisa	Dept. Reprod. Med. & Child Dev., Div. Obstet. & Gynecol.	Obstetrics Gynecology; Endocrinology Metabolism; Geriatrics Gerontology; Reproductive Biology; Cell Biology
4	Dr. Nanette Santoro	USA	60	31	50.60	3,036	254	Yeshiva University	Albert Einstein Coll. Med., Div. Reprod. Endocrinol.	Obstetrics Gynecology; Endocrinology Metabolism; Reproductive Biology; General Internal Medicine; Cardiovascular System Cardiology
5	Dr. Peter Chedraui	Ecuador	58	25	30.07	1,744	275	Catholic University of Guayaquil	School of Medical Sciences, Institute of Women’s Health	Obstetrics Gynecology; Geriatrics Gerontology; Endocrinology Metabolism; Urology Nephrology
6	Dr. Karen Matthews	USA	57	37	85.96	4,900	349	University of Pittsburgh	Dept. Psychiat. & Psychol.	Obstetrics Gynecology; Endocrinology Metabolism; Cardiovascular System Cardiology; Public Environmental Occupational Health; Neurosciences Neurology
7	Dr. Sebastian Mirkin	USA	57	18	22.00	1,254	142	Pfizer	Pfizer	Obstetrics Gynecology; Endocrinology Metabolism; Public Environmental Occupational Health; General Internal Medicine; Geriatrics Gerontology
8	Dr. Rossella Nappi	Italy	54	25	43.85	2,368	275	University of Pavia	Res. Ctr. Reprod. Med., Gynecol. Endocrinol. & Menopause Unit, Dept. Obstet. & Gynecol.	Obstetrics Gynecology; Geriatrics Gerontology; Endocrinology Metabolism; Urology Nephrology; Oncology
9	Dr. Howard Hodis	USA	51	26	50.25	2,563	324	University of Southern California	Keck. Sch. Med., Div. Cardiovasc. Med., Atherosclerosis Res. Unit	Obstetrics Gynecology; Cardiovascular System Cardiology; Geriatrics Gerontology; Neurosciences Neurology; Endocrinology Metabolism
10	Dr. Hadine Joffe	USA	51	32	54.04	2,756	476	Harvard University	Massachusetts Gen. Hosp., Dept. Psychiat., Perinatal. & Reprod. Psychiat. Clin. Res.	Obstetrics Gynecology; Endocrinology Metabolism; General Internal Medicine; Neurosciences Neurology; Psychiatry

ACPI, average citations per item; STC, sum of times cited; NCMCI, number of citations of most-cited items.

Dr. JoAann Manson possessed the highest number of publications (90), STC (8,169), ACPI (90.77), and H-index (41), followed by Dr. Karen Matthews in STC (4,900), ACPI (85.96), and H-index (37), Dr. Hadine Joffe (32) and Dr. Nanette Santoro (31) in H-index, Dr. Susan Davis (4,190) and Dr. Nanette Santoro (3,036) in STC. In addition, Dr. JoAann Manson (1,175) had the highest NCMCI, followed by Dr. Susan Davis (621), Dr. Hadine Joffe (476), and Dr. Karen Matthews (349) (Table 4).

### Analysis of hotspot

**FIGURE 8:**
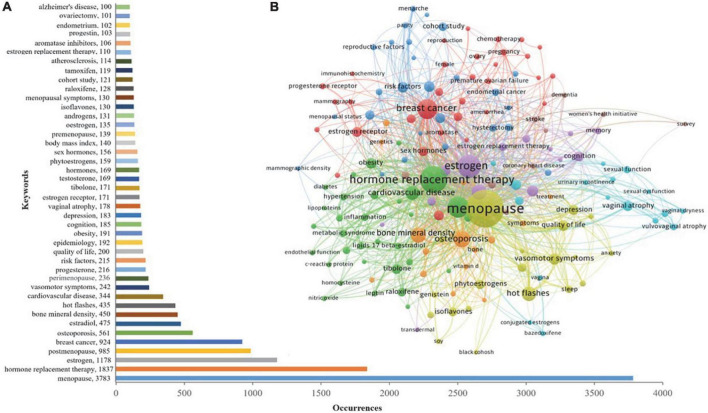
**(A)** The keywords of number of occurrences more than 100. **(B)** The network map of keyword clustering showed 165 keywords with a minimal occurrence of 30 times and classified into eight clusters.

A total of 12,863 keywords were identified from the 11,616 publications, and 165 keywords with occurrence frequency ≥ 30 were clustered. In Figure 8, one type of color represented one cluster, and the following eight clusters were developed: cluster 1 (yellow area, 14 items), research on the symptoms of menopause; cluster 2 (green area, 32 items), reporting the various types of MHT; cluster 3 (purple area, 14 items), published some estrogen receptor medicine; cluster 4 (red area, 32 items), studies on the breast and endometrium diseases; cluster 5 (orange area, 15 items), reported on bone density-related indicators; cluster 6 (blue area, 26 items), the epidemiology of the risk factors; cluster 7 (light blue area, 17 items), studies regarding sexual function; and cluster 8 (brown area, nine items), research on the women’s health (Figure 8B).

Next, we identified 42 keywords with a frequency ≥ 100. These keywords primarily focused on MHT, including estrogen (1,178) and estradiol (475), menopausal symptoms such as hot flashes (435), osteoporosis (561), and could be some adverse events of MHT such as cardiovascular disease (344), and cognitive impairment like the Alzheimer’s disease (100) (Figure 8A).

### High-frequent citation articles

Most of the top-10 most-cited publications came from the USA and were published in high impact factor journals such as JAMA, BMJ, and NEJM. Dr. Jacques Rossouw published the most frequently cited article (1,175), followed by Dr. Edward Bixler (1,000), Dr. Gillian Reeves (914), and Dr. Ethel Siris (884) (Table 5).

**TABLE 5:** The top-10 most-cited publications.

ID	Title	Country	Journal	Citations frequency
1	Post-menopausal hormone therapy and risk of cardiovascular disease by age and years since menopause (29)	USA	Journal of The American Medical Association (JAMA)	1,175
2	Prevalence of sleep-disordered breathing in women - Effects of gender (30)	USA	American Journal of Respiratory and Critical Care Medicine (AJRCMB)	1,000
3	Cancer incidence and mortality in relation to body mass index in the Million Women Study: cohort study (31)	USA	British Medical Journal (BMJ)	914
4	Identification and fracture outcomes of undiagnosed low bone mineral density in post-menopausal women - Results from the National Osteoporosis Risk Assessment (32)	USA	Journal of The American Medical Association (JAMA)	884
5	Palbociclib in Hormone-Receptor-Positive Advanced Breast Cancer (33)	UK	New England Journal of Medicine (NEJM)	873
6	Menopausal estrogen and estrogen-progestin replacement therapy and breast cancer risk (34)	USA	Journal of The American Medical Association (JAMA)	782
7	HPA-axis regulation at in-patient admission is associated with antidepressant therapy outcome in male but not in female depressed patients (35)	Germany	Psychoneuroendocrinology	664
8	Androgen levels in adult females: Changes with age, menopause, and oophorectomy (36)	Australia	Journal of Clinical Endocrinology & Metabolism	636
9	Transdermal testosterone treatment in women with impaired sexual function after oophorectomy (37)	USA	New England Journal of Medicine (NEJM)	622
10	A prospective population-based study of menopausal symptoms (38)	Australia	Obstetrics and Gynecology	615

## Discussion

Compared with the annual number of cardiovascular disease-related publications ranging from 3,276 to 9,836 in the past 20 years (39), MHT-related publications ranged from 438 to 650 annually, demonstrated a very slow increase. The research hotpots primarily focused on MHT for treating menopausal symptoms and the impact of MHT on women’s health. The most contributions to MHT-related publications were from the USA based on country, LERU in terms of the institution, and Dr. JoAann Manson from Harvard University, regarding the authors. By comparing the research fields of different countries, institutions and authors, which had the highest number of publications, we found that obstetrics gynecology and endocrinology metabolism had the most extensive research, followed by oncology and geriatrics gerontology in terms of country, oncology, public environmental and occupational health in terms of institution, and geriatrics gerontology in terms of author.

In the 1960s, estrogen therapy was invested and approved for treating menopausal symptoms, with very high popularity in the 1990s (40, 41). The first clinical trial, Women’s Health Initiative (WHI), focused on MHT and chronic post-menopausal conditions, started in the USA in the late 1990s (42). Prevalence of vasomotor symptoms alone is estimated at 40–50 million women in the USA, accounting for 38% among 1.2 billion women worldwide that will be menopausal or post-menopausal by 2030 (43). Based on the WHI that investigated about MHT and menopausal women, the USA became the country with the largest number of publications. After the announcement of the first results of WHI in 2002, MHT had more harms than benefits for health, then MHT usage was dropped (44). Simultaneously, our study showed a rapid decrease in MHT-related STC from 2002 (25,047) to 2003 (12,164) and ACPI from 2002 (45.62) to 2003 (22.74).

After that, MHT-related adverse events became the research hotspot. As shown in our study, the high-frequency keywords included breast cancer (924), cardiovascular disease (344), and cognition (185). However, the results from the clinical trials and observational studies regarding MHT adverse events remain inconsistent. On the one hand, the largest randomized controlled trial of MHT, WHI, which allocated post-menopausal women either to the placebo or the conjugated equine estrogen with medroxyprogesterone group, the latter depicted an increased 1.76 times risk of dementia (13) and 1.28 times risk of breast cancer (14). Additionally, the result showed 1.35 times more risk of stroke (15). On the other hand, some observational studies reported a reduced risk of Alzheimer’s disease and all-cause dementia in users of MHT (11, 16–18), and the use of exogenous estrogens were cardio-protective for post-menopausal women (10). Moreover, some randomized trials, such as the Danish Osteoporosis Prevention Study, observed that those receiving MHT showed a significantly reduced risk of the primary CVD composite endpoint compared to no treatment (HR: 0.48, 95% CI 0.26–0.87). The randomized trials named Kronos Early Estrogen Prevention Study and the Early Versus Late Intervention Trial with Estradiol have demonstrated a favorable safety profile of MHT when started early during menopause (45–47). Recent reviews attributed these inconsistencies to woman’s age, the duration of time since menopause, and different types and routes of administration (7, 12, 48). Therefore, results from the clinical trials and observational studies regarding MHT adverse events remain inconsistent. Thus, evidence of mechanisms can be decisive, enabling researchers to explain, predict, and intervene (19). Additionally, our study showed that the evidence regarding mechanisms was limited, so mechanistic research could be a potential research hotspot.

## Strength and limitations

Since this was the first bibliometrics analysis of MHT-related publications, our study provided a good insight into research hotpots, exposed the evidence gap, and provided research ideas for researchers. In this bibliometric analysis, we also explored the top-5 research areas of the top-10 countries and top-10 authors.

However, our study had certain limitations. Firstly, although most MHT-related research manuscripts were indexed in the WOS database, our study could have omitted some important studies (49–51). Secondly, only English documents were included in our analysis based on the inclusion criteria. Thus, some essential non-English studies might have been excluded from the assessment. Thirdly, we selected the study types through the filters in the WOSCC. A small number of meta-analyses may index as articles. Some of the data we analyzed were automatically extracted from the downloaded publications by the software, like the authors’ names. The software could not distinguish between authors with the same name, which might affect the results of our study.

## Conclusion

Menopausal hormone therapy (MHT)-related publications revealed a very slow increase trend. The USA played a vital role in MHT-related studies. Moreover, Dr. JoAann Manson from the University of Harvard had the most contributions. The research topics primarily focused on treating menopausal symptoms and the impact of MHT on women’s health. Results from the clinical trials and observational studies regarding MHT adverse events remain inconsistent. To clarify this inconsistency, more researchers should focus on adverse events and their mechanisms.

## Data availability statement

The datasets presented in this study can be found in online repositories. The names of the repository/repositories and accession number(s) can be found in the article/Supplementary material.

## Ethics statement

Ethical review and approval was not required for the study on human participants in accordance with the local legislation and institutional requirements. Written informed consent for participation was not required for this study in accordance with the national legislation and the institutional requirements.

## Author contributions

KY, JL, ZW, and CQ led the team, were responsible for all aspects of the project, and contributed to the conception and design of the work. JL and ZW were in charge of the literature search and data acquisition. JL, ZW, JW, KM, XL, YY, YL, NZ, AS, JH, and CQ collected, analyzed, and interpreted the data. JL, ZW, JW, KM, XL, and YY wrote the first draft of the manuscript. JL, KY, and CQ were responsible for the editing and standardization of the tables and figures and gave critical advice on the manuscript. All authors contributed to the manuscript revision, read, and approved the submitted version.
