# Supplementary material for: Trends in research related to menopausal hormone therapy from 2000 to 2021: A bibliometric analysis
Source: Front Med (Lausanne). 2022 Oct 28;9:952487. doi: 10.3389/fmed.2022.952487 (PMC9649926; doi:10.3389/fmed.2022.952487)
Supplement: Supplementary file 1 [file Table_1.docx]

Supplementary Table 1. Search strategy.

| **#1** Hormone Replacement Therapy (TS) or Estrogen Replacement Therapy (TS) or Estrogen* (TS) or Progestin* (TS) or Medroxyprogesterone (TS) or hormone therapy (TS) or menopausal hormone therapy (TS) or HRT (TS) or ERT (TS) or Progestins (TS) or medroxyprogestrone acetate (TS) or dydrogesterone (TS) or norethisterone (TS) or norethindrone (TS) or oestrogen (TS) or estrogen (TS) or conjugated equine estrogen (TS) or CEE (TS) or Premarin (TS) or Estriol (TS) or Oestradiol (TS) or estradiol* (TS) 350,842 |
| --- |
| **#2** Menopause (TS) or Climacteric (TS) or Postmenopause (TS) or post-menopaus* (TS) or post menopaus* (TS) or perimenopausal (TS) or Premenopause (TS) or perimenopause (TS) 49,001 |
| **#3** #1 AND #2 22,013 |
| **#4** rat* (TI) or mouse (TI) or mice (TI) or animal* (TI) 2,407,141 |
| **#5** #3 NOT #4 20,274 |
| **#6** #5 AND 2000 or 2001 or 2002 or 2003 or 2004 or 2005 or 2006 or 2007 or 2008 or 2009 or 2010 or 2011 or 2012 or 2013 or 2014 or 2015 or 2016 or 2017 or 2018 or 2019 or 2020 or 2021 [published year] 15,976 |
